# Causal Loop Analysis Can Identify Solutions to Complex Dog Management Problems in Remote Australian Aboriginal Communities

**DOI:** 10.3390/ani11041056

**Published:** 2021-04-08

**Authors:** Brooke P. A. Kennedy, Wendy Y. Brown, James R.A. Butler

**Affiliations:** 1 School of Environmental and Rural Science, University of New England, Armidale, NSW 2353, Australia; wbrown@une.edu.au; 2 CSIRO Land & Water, GPO Box 2583, Brisbane, QLD 4001, Australia; james.butler@csiro.au

**Keywords:** animal management, stakeholder participation, causal loop analysis

## Abstract

**Simple Summary:**

Population and health management of dogs and cats in remote communities is challenging due to limited access to veterinary services and high reproductive rates. Financial support for companion animal management within communities is limited and programs delivered by external providers rarely achieve sustainable outcomes. An alternative approach, whereby community participation is enlisted from the beginning before implementing any interventions, may help to achieve sustainable improvements in animal populations and to improve animal health. To this end, stakeholders were interviewed and it was determined that dog overpopulation was the overarching issue. Focus group discussions were then conducted with three of the four same stakeholder groups to uncover the main causes of this issue, followed by solutions being generated that the community could undertake to address the issue. Similar causes were discussed and multiple solutions were developed, with education and training prioritised as the top solutions by all three groups. These will require transformational social adaptations to build the capacity of the local community to implement the solutions.

**Abstract:**

Companion animal management in Australian remote Aboriginal communities (rAcs) is a complex problem with multiple stakeholders involved, with differing needs, knowledge, power and resources. The Comm4Unity (Cycle of Multiple Methods for Unity—For Community) approach was designed to address such problems. This study represents the second step of the Comm4Unity framework, where a causal loop analysis (CLA) was adapted and tested as a tool to address the issue of dog overpopulation in Wurrumiyanga, and in particular the systemic causes of the problem and necessary transformational management solutions. Ten focus group discussions (FGDs) were held amongst three of the four stakeholder groups identified during the first step in the analysis. The CLA identified 13 positive feedback loops, which drive vicious cycles and perpetuate the dog overpopulation issue. All three groups agreed and developed 22 solutions to address the causes of dog overpopulation. Despite the differences in the framings of the three groups, “training” and “education” were both the top priority solutions for all three groups. The majority of the solutions discussed by the groups were not only transformational but also social, requiring collaboration. This study was successful in so far as transformational actions were co-developed by all FGDs, which may have also built capacity and agency amongst the local community to implement them as a cohesive group.

## 1. Introduction

The world is increasingly beset by complex problems, caused by escalating technical, social and economic connectivity, which drive and are driven by rapid globalisation. These complex issues have both content and process complexities, and the more “wicked” they become, the harder they are to solve [1]. Complex content describes multidimensional problems with differing aspects joining together under the one issue, making the problem difficult to define, whilst process complexity refers to issues where an elaborate network of stakeholders are involved with differing values and goals [2]. These are multifaceted problems that cannot be solved by one person or group, and therefore require multiple stakeholders to input their diverse knowledge and generate solutions [2]. Ideally, to achieve sustained outcomes and impacts, participatory methods should be applied, such as collective or social learning [3], which can “shift the burden” from short-term solutions focused on symptoms to longer-term solutions by addressing the root causes of problems [4,5].

Animal management is often characterised by such complex problems. For example, “conservation conflict” is driven by conservation groups wishing to protect wildlife species that impact the livelihoods of others, polarising the stakeholders involved and causing intractable, encultured conflict [6,7]. The colonial, racial and cultural dynamics of human–animal relations need further investigation [8], however a recent study in Romania utilised more-than-human (in this case, dog) geographies to critically review the divided public opinion on the rapid implementation of a street dog law [9]. Similarly, companion animal management may cause conflict for the mere fact that companion animals are involved. Companion animals, particularly dogs, have strong geographies, but they are also involved in other areas, including as laboratory subjects, where they have been successful models for multiple human processes [10,11]. Companion animals may also negatively impact human health and wildlife conservation, for example amongst free-ranging dog populations (e.g., [12,13,14,15]). Resolution of such conflicts, whilst maintaining ethical and culturally appropriate management methods, requires stakeholder values and goals to be addressed via equitable participation, collaborative decision-making, problem identification and resolution [16,17].

**Figure 1:**
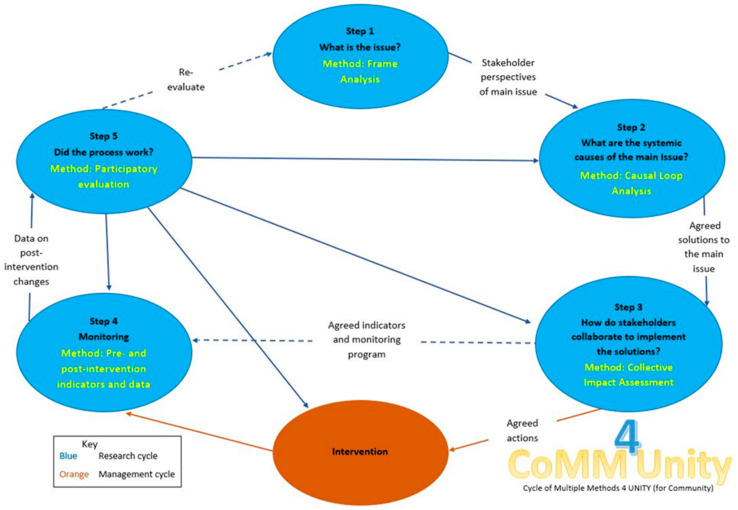
The Comm4Unity framework [31].

In northern Australia, companion animal management is posing a growing problem. Domestic free-roaming dogs and cats in remote Aboriginal communities (rAcs) pose health risks to humans [15,18,19,20] and threaten local wildlife [21,22,23,24]. They are also likely to become a primary vector of rabies transmission to humans and wildlife populations should the virus enter Australia [25]. Dingoes and dogs have been an integral part of Aboriginal families for decades [26]. The rapid transition from dingoes to domestic dogs as companions and from nomadic to permanent lifestyles post-European colonisation are contributing factors to the poor environmental health standards of many Aboriginal communities [27], poor dog health from overcrowding and an inability to feed large numbers of dogs [26]. Tough dog restrictions in the past were enforced by authority figures who could lawfully destroy any dog that was deemed to not meet the imposed criteria [28]. These factors are commonly thought to be reasons why animal management programs have failed in Aboriginal communities in the past [26]. Cats have only recently been introduced to many rAcs [29], so there is not a history of tension between community members and authority figures in relation to cat ownership, however prior negativity towards authority figures in relation to companion animal management generally is most likely still at play. A previous frame analysis identified that in one rAc, namely Wurrumiyanga in the Tiwi Islands, four distinct stakeholder groups existed with differing perspectives and goals for dog and cat management [30]. Whilst some of their views overlapped, each group had distinct knowledge cultures and power frames. A common theme mentioned by all four groups, however, was dog overpopulation. This analysis was the first step in a practice-based approach, the Comm4Unity (Cycle of Multiple Methods for Unity—For Community) framework (Figure 1) [30], whereby multiple stakeholders’ knowledge is engaged and integrated to generate collective action in an adaptive learning cycle [31]. There are scant data available in the scientific literature on the issue of dog overpopulation, and specifically the systemic causes of the problem and necessary transformational management solutions. This paper describes the second step, whereby the causes of the overpopulation of dogs are analysed from a systems perspective and priority management solutions are collectively identified.

Causal loop analysis (CLA) is a participatory process that enables stakeholders to identify and understand the sources or root causes of a problem and the relationships between them, rather than the symptoms that they create [32]. Causal loop analysis differentiates the direct and indirect impacts of a problem then identifies its direct (proximate) and indirect (systemic) causes [33]. More importantly, by applying systems principles, CLA can include the causal feedback loops that link the impacts to the causes of the problem; positive (or reinforcing) feedback loops amplify the problem, whilst negative (or balancing) loops dampen the effects [34]. Although CLA has been tested in various natural resource management contexts [34,35,36,37], it has not been applied to dog and cat management in Australian rAcs. The specific features of Indigenous natural resource management, which involve distinctive local knowledge and acute power asymmetries between the government and communities, may provide an opportunity to test and adapt CLA as a participatory technique that can bridge these gaps. In this paper, which represents the second step of the Comm4Unity framework, we adapted and tested CLA as a tool to address the issue of dog overpopulation in Wurrumiyanga, and in particular the systemic causes of the problem and necessary transformational management solutions.

## 2. Materials and Methods

### 2.1. Study Site

The Tiwi Islands are made up of 9 uninhabited islands and 2 inhabited islands located 60 km off the coast of Darwin, Northern Territory, along the northern coastline of Australia. The main two islands are mostly uninhabited, with most residents living in one of the three main communities: Milikapiti (401 residents) and Pirlangimpi (371 residents) on Melville Island and Wurrumiyanga (1563 residents), the capital, on Bathurst Island [38].

This research focuses on the community of Wurrumiyanga and their companion animals. The dog population of Wurrumiyanga was estimated at 326 ± 52 in 2014 [39]. A similar estimation of 343 dogs was reported in Wurrumiyanga in 2017 and an increase in domestic cat ownership was also noted [29]. Animal Management in Rural and Remote Indigenous Communities (AMRRIC) is a non-profit charity established to conduct companion animal health programs in rAcs in Australia [40,41]. AMRRIC assist in conducting these dog health programs in Wurrumiyanga by recruiting volunteers and providing resources when needed and available, including parasitic medications and educational resources and staff. Two of the authors (B.K. and W.B.) have participated in multiple dog health programs in Wurrumiyanga since 2013 as volunteers and researchers.

Although no level of government in the Northern Territory formally holds the animal management portfolio, in the absence of veterinary services on the islands, the local council, Tiwi Islands Regional Council (TIRC), have contracted a veterinary service for the Tiwi Islands twice a year for periods of one week for the last two decades. The same veterinarian has been contracted for most of these services for the last 22 years, over which time he has gained the trust of the Tiwi locals. However, other governance has sometimes dampened the relationships between locals and animal management strategies. For example, as recent as 2012, a different veterinarian was contracted for a single program, specifically to conduct a cull of dogs, during which hundreds of dogs were killed with no regard for their owners or the cultural or companion relationships they had with them. New by-laws for keeping dogs were recently introduced (2018), although have not yet been enforced, outlining mandatory dog registration and a restriction of two dogs per household [42]. The TIRC has also introduced a new fee, whereby community members need to pay $50 to receive veterinary services (de-sexing and parasitic medication) for up to two dogs to help cover the cost of contracting the veterinarian [43].

### 2.2. Participant Engagement

Engaging stakeholders in rAcs is challenging due to the wide dispersal of government versus local community members, limited communications infrastructure and logistical constraints [29]. Despite this, in the previous step of the frame analysis [29], four stakeholder groups with differing goals and values regarding companion animal management in Wurrumiyanga were identified: indigenous locals (IL), indigenous rangers (IR), animal managers (AM) and non-indigenous locals (NIL). Indigenous locals believed that dogs are a bigger problem than cats and that there are too many. As dogs have strong cultural connections to the Tiwi people, the IL considered that any animal management program should be culturally appropriate. Indigenous rangers are responsible for environmental management, including feral animals outside the community borders, and therefore undertake animal management activities across all species. They considered that stakeholders should work together to create and enforce policies to achieve this. Although many domestic, feral and wild species were discussed, dogs and their numerous impacts, and overpopulation in particular, featured most regularly. Animal managers also agreed that there were too many dogs and were also concerned about the increasing number of cats. They considered that dog management must be intensified to improve animal and human health, and securing funding and building the capacity of local inhabitants were their main goals. Non-indigenous locals were also concerned about the large number of dogs and felt that reducing numbers would not only improve the possibility of ensuring a healthy dog population, but also reduce human health risks.

Because they had differing knowledge about animal management and there was a power asymmetry amongst them, the CLA was originally intended to be carried out with representatives from each group separately using focus group discussions (FGDs). This would have allowed the identification of their common and diverging diagnoses of the systemic causes of dog overpopulation and their differing priorities for solutions and management actions. Members of the four stakeholder groups engaged in the previous step were approached to be involved in the CLA. Any persons involved in animal management (stakeholder) in Wurrumiyanga that fell into one of these four groups were eligible for involvement. The AM and IR groups agreed to participate (Table 1), however although multiple attempts at contact were made, no NIL group members responded. Since the IL group involved local community members, they were approached via the leader’s forum (a group of four local Tiwi people, each representing one of the four “skin groups” of the Tiwi Islands), who agreed to participate in the first FGD. After the exercise, the group of four skin group leaders were asked for permission for other local community members to participate, and whether the process should be modified to be more useful. All of the skin group representatives agreed that the proposed process would be acceptable as planned if the participation information sheet was presented verbally at the beginning of an FGD (which was necessary to comply with the CSIRO Human Research Ethics approval, 137/17), the process was explained and anonymity was guaranteed for all participants. While they agreed that FGDs did not have to be held with only one skin group, participants segregated themselves voluntarily into men and women, as is customary for their culture (Table 1). Additionally, FGDs were held in a location in Wurrumiyanga where participants felt comfortable and able to discuss issues freely.

### 2.3. Causal Loop Analysis

Causal loop analysis was first described as a system of closed boundaries and feedback loops by Forrester [44] to illustrate a dynamic system, not only in terms of individual system components, but also their interconnections. It was applied to the planning of public works by Maruyama [45], who emphasised that the cultural, social and psychological characteristics of all stakeholders should also be engaged. Causal loop analysis has been applied in various different contexts to explore solutions to complex problems, for example in business [46], watershed management [34] and rural development [32]. When carried out as a multistakeholder participatory process, it enables the integration of different knowledge types or cultures to understand and then design innovative solutions [33], often resulting in a reprioritisation of policy and funding needs [47]. Based on such an analysis, it is possible to identify and priortise critical interventions that will address systemic causes and break positive feedback loops that are creating “vicious cycles”. Incremental solutions can tackle direct proximate causes, whereas transformational actions address the underlying systemic causes that are necessary to create significant progress in solving the problem [32,48].

The CLA method was adapted from Butler et al. [32], which combines the Stockholm Environment Institute (SEI) and the Centre for International Forestry Research [49] problem tree tools with systems thinking and feedback loops [33]. Butler et al.’s [17] process follows four steps. Step 1 identifies the direct and indirect impacts emanating “downstream” from the problem and the causal linkages between them. Step 2 identifies the direct and indirect causes “upstream” of the problem and the linkages between them. Step 3 identifies the primary feedback loops from the impacts to the causes, which either amplify (positive feedback) or dampen a cause (negative feedback). Step 4 identifies priority actions and solutions to address the causes. Designing solutions that target feedback loops is important, because otherwise the feedbacks can maintain a “vicious cycle”, perpetuating the problem. The solutions are ranked according to participants’ consideration of their importance in addressing systemic causes and in breaking feedback loops and vicious cycles. Those solutions considered most transformational are ranked highest.

**Figure 2:**
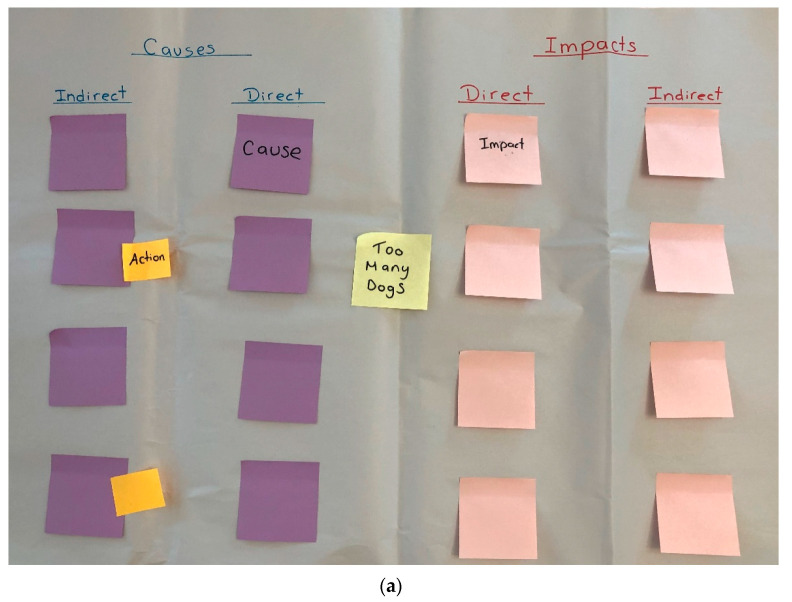

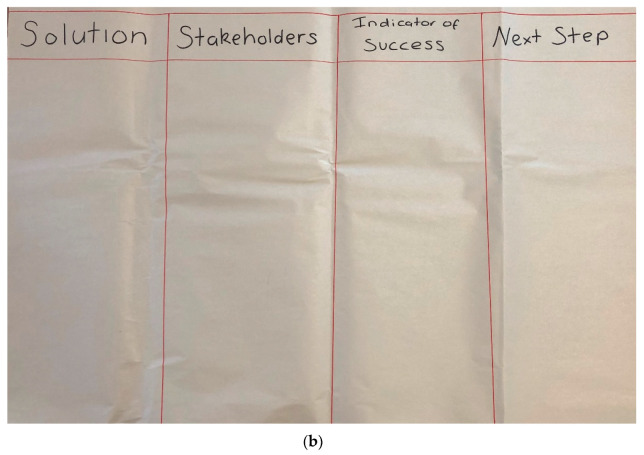
Pre-drawn (**a**) CLA template and (**b**) solutions table template used during FGDs.

The equipment used in the FGDs was simple: butcher’s paper used for drawing the CLA; sticky notes (Post-it^®^, Cynthiana, KY, USA) (four different colours) for the issue, impacts, causes and solutions; blu-tack (Bostik, Middleton, MA, USA) to attach the CLA diagrams to the wall for easy viewing by participants; as well as black and red permanent marker pens and clear adhesive tape. Before the FGDs, 15 CLA templates (Figure 2a) and 15 solution tables (Figure 2b) were prepared on butcher’s paper to save time on the day. One colour (yellow) of the sticky notes was used to write down the central “issue” (i.e., “too many dogs”) in the middle of the butcher’s paper. A second colour (pink) was used to record both the direct and indirect impacts. A third colour (blue) was used to record both the direct and indirect causes. All writing was in black marker. Once feedback loops had been determined, they were drawn and annotated in red marker. A fourth colour (green) of sticky note was then used to add the solutions to the butcher’s paper (Figure 2a).

### 2.4. Solutions

After solutions had been identified in each FGD, participants filled in the table of solutions (Figure 2b). The first column, “solution”, listed each solution in descending order of priority from the CLA. The second column, “stakeholders”, nominated the people or organisation(s) that the group considered best-placed and responsible for actioning the solution, considering their power and agency. The third column, “indicator of success”, detailed the process required to measure how the solution had been implemented and whether it was successful. The fourth column, “next step”, identified the immediate steps that the FGD participants had to undertake to instigate the solution, including individuals that participants should ask or speak to in order to catalyse progress. It was then discussed whether the actions deliberated were already happening, had been discussed and was in the process of starting, or had only been discussed but had not proceeded or had not been discussed at all. The CLA diagrams and solutions tables were later recreated electronically for visual ease of analysis.

## 3. Results

### 3.1. Causal Loop Analysis

**Figure 3:**
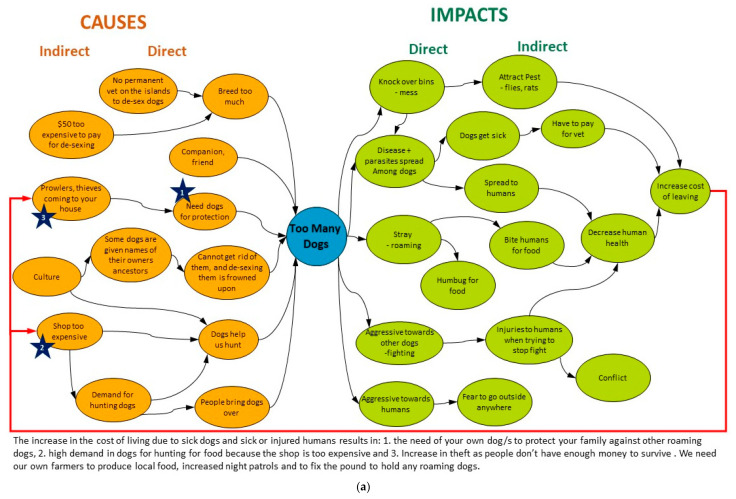

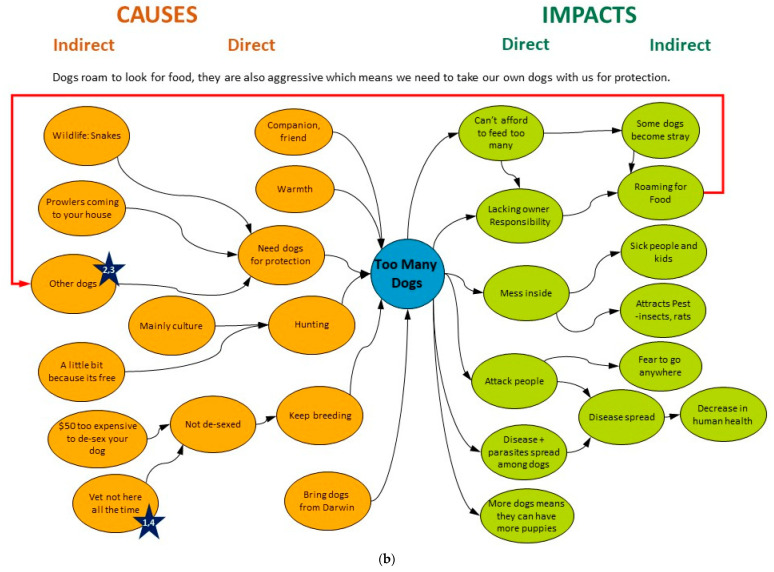

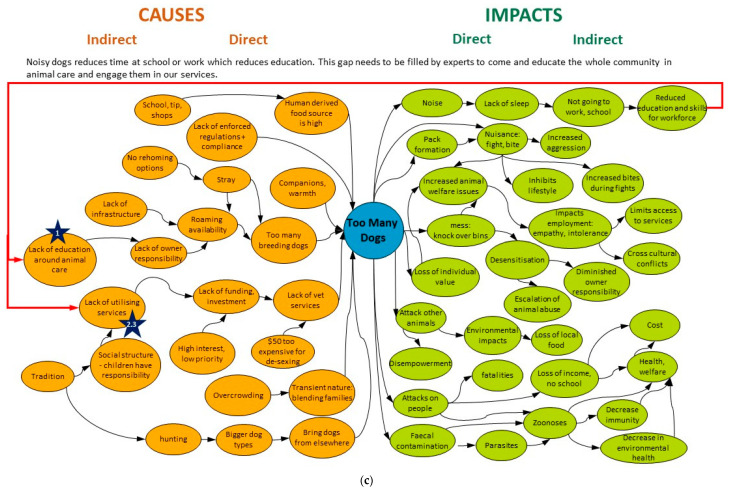
Causal loop analyses conducted in focus group discussions in Wurrumiyanga. The pre-determined animal management problem of “too many dogs” (i.e., dog overpopulation) was discussed by (**a**) indigenous locals (IL, Appendix A), (**b**) indigenous rangers (IR) and (**c**) animal managers (AM).

**Figure A1:**
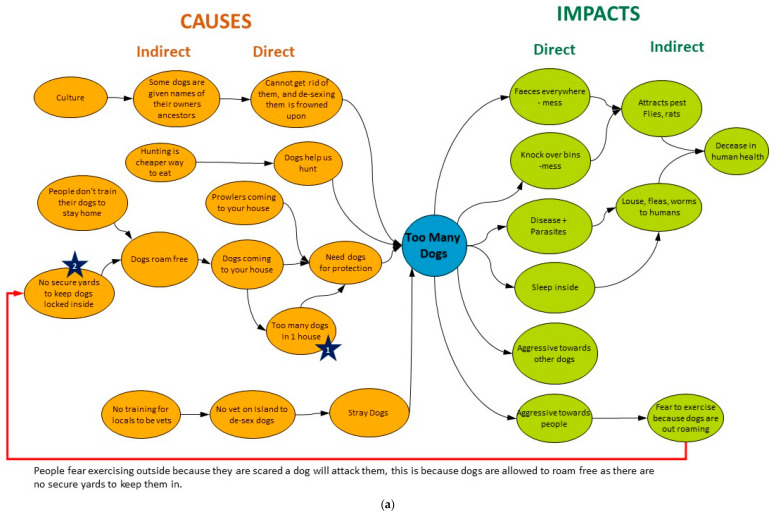

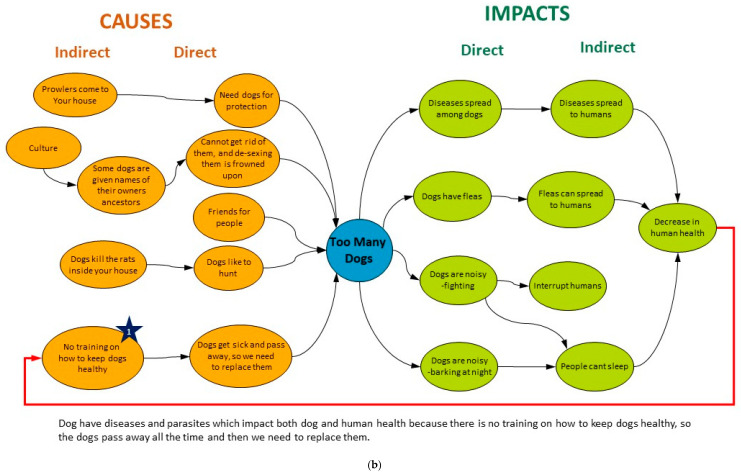

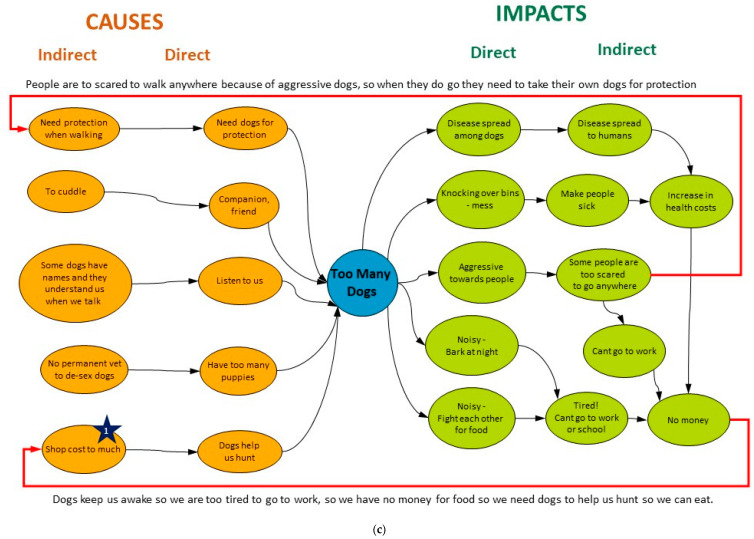

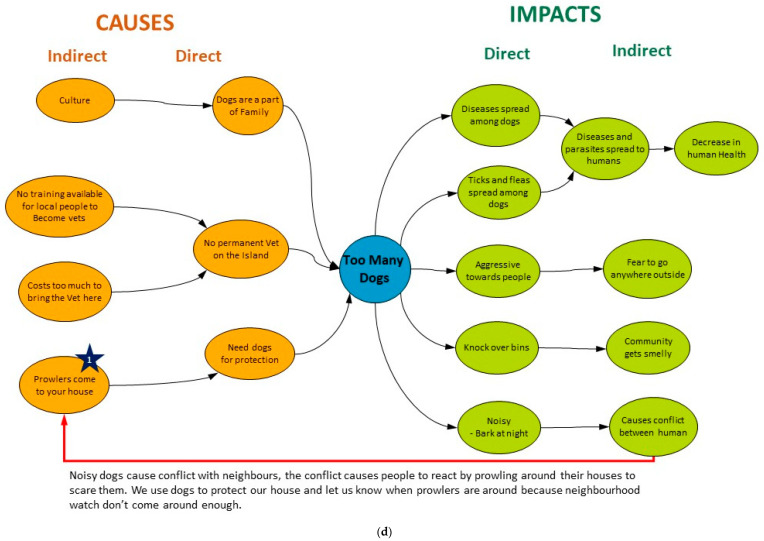

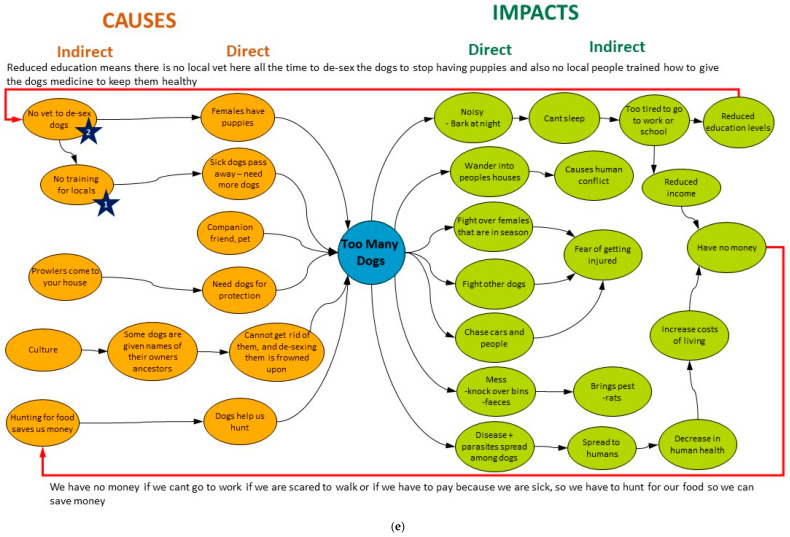

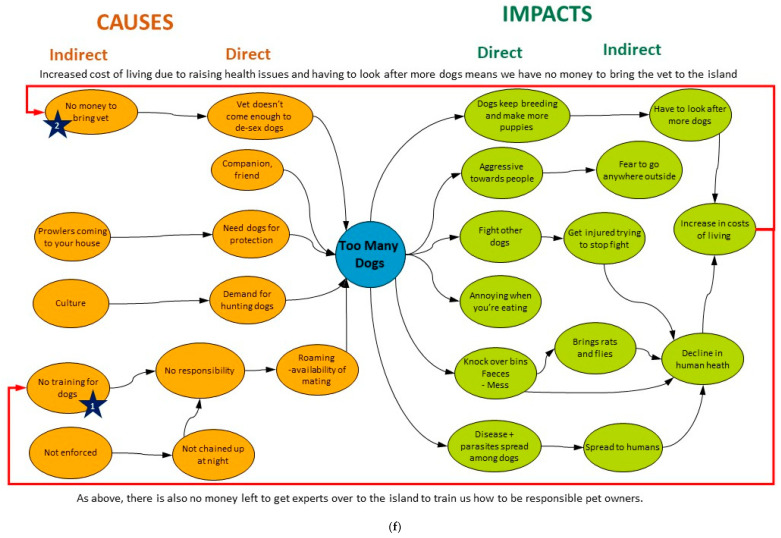

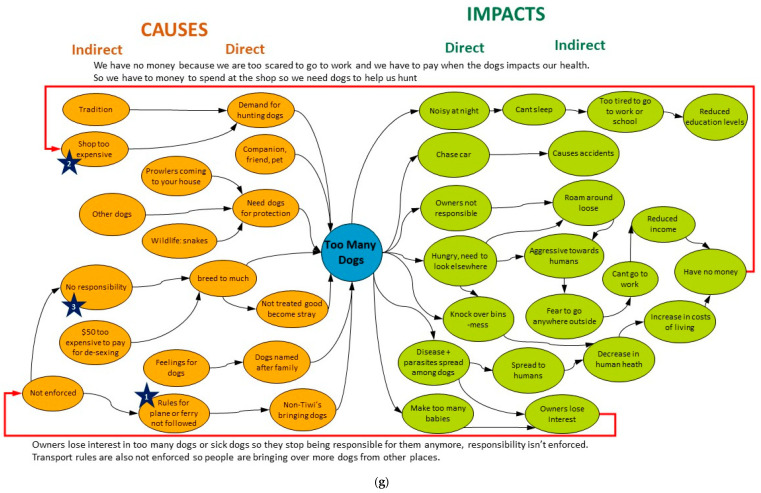
Causal Loop Analysis (CLA) of ‘too many dogs’ conducted during Focus Group Discussions (FGDs) with Indigenous Locals (ILs) of Wurrumiyanga. The first seven CLA’s conducted in FGDs with ILs are presented in (**a**–**g**), the eighth CLA with ILs, and the single CLA by IR, and AM groups can be found in the main text.

Summaries of the CLAs from the 10 FGDs are presented below. Examples from the three stakeholder groups represented are shown in Figure 3 (the remaining CLAs are presented in Appendix A Figure A1).

#### 3.1.1. Indigenous Locals

##### Focus Group 1

This focus group identified one positive feedback loop that focused on the fact that dogs roam freely throughout Wurrumiyanga (Figure A1a). Although it was suggested as a part of responsible pet ownership that dogs should remain at home, this group suggested two reasons why they do not. Firstly, some houses have too many dogs and they cannot control them, so the dogs roam free. The second was that even if they wanted to restrict them to the home, there is no secure fencing to enable this. Some yards do have fences, but the dogs dig underneath them. These roaming dogs are sometimes aggressive towards people, especially when they congregate in groups. This creates fear amongst residents, especially those that wish to exercise but cannot because they are chased by dogs. The primary solution was an incremental action, the laying of cement underneath fences to stop dogs from digging out. The second solution, a transformational action, was the introduction of a two-dog by-law to reduce the number of dogs per household.

##### Focus Group 2

This focus group identified one positive feedback loop that focused on the health impacts of having too many dogs (Figure A1b). Dogs spread diseases and parasites amongst each other because they roam together all day. They also roam where people live, and so diseases and parasites can also “spillover” to residents. This affects people’s health, and hence they sometimes cannot go to school or work. The group believed that this is caused by a lack of training for dog owners about how to keep dogs healthy. As a result, dogs become ill and their owners stop looking after them, resulting in more stray dogs or the dogs dying. Either way, the owners replace the dog with another, resulting in dog population growth. The group suggested that the priority solution was a transformational action, the provision of more training to educate owners about looking after their dogs in order to reduce the spread of parasites and disease amongst dogs to people.

##### Focus Group 3

This focus group identified two positive feedback loops, although a solution was only recommended for one (Figure A1c). The first loop was similar to that discussed by FGD1, whereby there was a fear amongst residents of moving around the settlement because of roaming dogs. However, this group focused on the fact that as a result they needed to obtain more dogs to protect themselves from the roaming dogs. This led to the second feedback loop, whereby households that do not obtain their own dogs for protection cannot leave the house to work, and so do not earn any income. This feedback loop also examined other reasons why people can or cannot work, including problems such as barking dogs, which keep them awake at night and consequently make them too tired to work, meaning they do not get paid. This results in people obtaining more dogs so that they can hunt to source wild food as a substitute for supermarket-bought food, which they cannot afford with limited income. This problem is exacerbated by the fact that supermarket food is expensive because it is imported. This FGD recommended one transformational solution, namely the initiation of a community farm to produce local food that will be cheaper than the imported supermarket food.

##### Focus Group 4

This focus group identified one positive feedback loop that focused on dogs barking at night, which keeps everyone awake and causes conflict between households (Figure A1d). This causes people to confront each other or to attack the offending dog. As a result, people acquire more dogs to protect their homes from prowlers. This group recommended a transformational solution, an increase in the number of neighbourhood watch staff and patrols to protect houses from prowlers.

##### Focus Group 5

This focus group identified two feedback loops that stemmed from the same direct impact of dogs barking at night, which resulted in a lack of sleep and fatigue, preventing them going to work or school (Figure A1e). The indirect impact of reduced income initiated the first feedback loop, leading to hunting for food to save money. This results in people acquiring more dogs to assist with hunting, which exacerbates the dog overpopulation problem. No solution was recommended for this loop. The indirect impact of reduced education levels due to fatigue caused by noisy dogs initiated the second feedback loop, leading to no veterinarian being present on the island because locals are not sufficiently educated to understand the importance of de-sexing dogs, which results in large numbers of puppies. The limited education levels also result in a lack of local people being adequately trained to administer dog treatments between the veterinarian’s visits. Two transformational actions were recommended to tackle this feedback loop: the first priority was to train local people in animal management to improve dog health, while the second was to increase the number of veterinarian visits to the island.

##### Focus Group 6

This focus group took many factors into account that led to indirect impacts of increased costs of living due to the expense of looking after more dogs and the cost of health services due to their effects on human health (Figure A1f). These increases in living costs result in the community having limited funds, and therefore there are limited veterinarian visits and other experts cannot be afforded to train local people in responsible dog husbandry. This group recommended that two transformational actions were necessary: first, responsible dog ownership should be introduced into the schools with expertise brought from the mainland; and second, local people should be trained in animal management to improve dog health and reduce population growth. As a consequence, veterinarian visits would be required less often and the current rate of two visits per year would be sufficient.

##### Focus Group 7

This focus group also took many factors into account that result in the indirect impact of households having little cash (Figure A1g). This group identified two positive feedback loops. The first was not being able to work because of the fear of being attacked by roaming dogs outside their homes, which means that they earn no income with which to buy the high-priced supermarket food, and as a result they need hunting dogs to source subsistence food. The second loop involved owners losing interest in their sick dogs because other dogs were having more puppies. Consequently, owners give up responsibility for their sick dog, which then becomes a stray and breeds freely, producing more dogs, increasing the dog population. The disappearance of one dog then leaves room for a new dog, and because of a lack of enforced rules, people can obtain new dogs without consequence, again increasing the dog population.

This group recommended three transformational actions. First, the community must introduce and enforce rules that restrict the importation of dogs to the island. Second, local food production must be promoted to replace the expensive imported food. Third, education and awareness about responsible pet ownership must be improved and rules must be enforced to deter irresponsible husbandry.

##### Focus Group 8

This focus group’s perspectives mirrored those from the other IL FGDs, highlighting the increasing cost of living in Wurrumiyanga (Figure 3a). This is driven by the cost of removing pests, which have been attracted to waste bins knocked over by dogs, veterinarian costs for dog treatment when they contract diseases and parasites from roaming dogs, as well as health service costs incurred when people need treatment for zoonoses and injuries caused by aggressive roaming dogs. The first feedback loop resulting from these rising costs was that no one can afford veterinary treatment, and hence de-sexing dogs is rare, escalating dog population growth. The second was that shop-bought food is too expensive and owners need hunting dogs to supply subsistence food. The third was that people are driven to theft because they have no money, and hence households are obtaining more dogs for protection. Three transformational actions were recommended. First, the TIRC dog pound must be repaired to enable the removal of any stray dogs from the community and reduce the number of breeding females. Second, the community must establish farms to grow their own food. Third, the number of police and neighbourhood watch patrols must be increased, especially at night, to deter theft and robbery and reduce the need for guard dogs.

#### 3.1.2. Indigenous Rangers

##### Focus Group 9

Although this focus group only identified one positive feedback loop, it encompassed many of the issues discussed throughout their analysis (Figure 3b). It was agreed that when there are too many dogs it is hard to feed them, and so dogs roam to look for food, some of which are aggressive, resulting in local households needing their own dogs for protection. The group discussed four transformational actions covering a range of issues stemming from these causes. In order of priority, the group first recommended engaging local inhabitants in animal management through training. Second, teaching owner responsibility in schools is necessary to reduce the number of roaming dogs. Third, new laws should be created to limit the number of dogs owned per household, and therefore the overall population. Fourth, more veterinarian visits to the community are required, however collaboration with other partners is necessary to access more funds.

#### 3.1.3. Animal Managers

##### Focus Group 10

This focus group’s analysis was more comprehensive than the others, covering all of the impacts discussed by the others, plus additional ones (Figure 3c). The group identified two positive feedback loops beginning from a third-order indirect impact, the lack of education and skills among the Tiwi workforce. The first loop emanating from this impact resulted in a lack of education about how to care for animals overall. The second loop identified some Wurrumiyanga residents’ inability to utilise animal health services. The group discussed three transformational actions. The top priority is the need to deliver “responsible dog ownership” programs to empower local people. The second is to utilise expert services to train local people in parasite control to improve dog health. The third is for the community authorities to include external experts when implementing new solutions regarding animal management.

#### 3.1.4. Summary

A total of 13 positive and no negative feedback loops were identified. Four of the loops created by the ILs were focused on families obtaining hunting dogs to assist with hunting for food because food purchased from the shop is too expensive for those experiencing increased living costs. The ILs also had two loops focused on families having dogs to protect their homes from people prowling, and another two focused on having dogs to protect them from aggressive dogs in the community. This mirrored the IRs, whose single feedback loop focused on dogs used for protection against other dogs that are free-roaming in the community. Eleven loops were addressed through 20 transformational and two incremental actions. Two feedback loops were not addressed due to a lack of time (one each from FGDs 3 and 5, both with ILs).

### 3.2. Solutions

Due to the similarities amongst groups with regards to the solution(s) they identified, a combined solutions table was created (Table 2). Training and education were the top two priority themes, followed by the establishment of local food production, the introduction of by-laws to control dog numbers, increased night patrols to deter theft and increased numbers of veterinarian visits (Table 2). Of the solutions, 19 had not yet been initiated, three were beginning and none were underway or complete.

Each of the main themes of the solutions in Table 2 are presented in Table 3. The most frequently mentioned was “training”, closely followed by “education”. All three of the stakeholder groups mentioned solutions in these two themes. Additionally, all of these actions were transformational, tackling the indirect, systemic root causes of the dog overpopulation problem.

## 4. Discussion

Complex problems are characterised by different stakeholder groups and their knowledge, as well as their conflicting goals and values. Wicked (as opposed to tame) problems are those with multiple definitions given by different stakeholders with differing needs, meaning that there is no true resolution, and therefore conflict escalates over a persistent and intractable issue, often perpetuating the issue [1]. Therefore, they require multiple stakeholders to contribute their diverse knowledge and solutions [2], ideally using participatory processes to focus on the root causes of problems [4,5] in an attempt to transform the system rather than simply maintaining its current form [50].

We held FGDs with three of the four stakeholder groups identified in the frame analysis in the first step of the Comm4Unity framework [29], who coalesced around the common problem of dog overpopulation. Many of the impacts discussed have been reported in other rAcs. For example, FGDs 1–9 all discussed the transmission of diseases or parasites from dogs to humans. For example, Smout et al. [15] observed the presence of hookworm (*Ancylostoma ceylanicum*) in 22% of dogs and 56% of soil samples in a northern Australian Indigenous community. Five of the eight IL FGDs and the IR FGD also discussed that locals have dogs to help them hunt for food, typically because shop-bought food is too expensive. This has also been reported elsewhere, for example by Barber et al. [51], who highlighted the importance of subsistence fishing and hunting for food security in rAcs across northern Australia.

The CLAs identified 13 feedback loops. None were negative, meaning those that would dampen the system effects. Instead, all were positive, reinforcing the vicious cycles and perpetuating the wicked problem of dog overpopulation. All three groups agreed to the dog overpopulation issue and collectively developed 22 solutions (20 transformational and two incremental) to address the causes of having too many dogs. Despite the differences in the framings of the three groups, “training” and “education” were both the top priority solutions for all three groups. Capacity building, such as in training and education, is crucial to enable transformational action [52] to the extent that the term “transformative capacity”—the capacity of individuals and organisations to imagine, enact and sustain a transformed society in a deliberate way [53]. Transformation, through the building of relationships and the co-development of solutions, is necessary if wicked problems are to be tackled [52]. The majority of the solutions discussed by the groups were not only transformational but also social, requiring collaboration, linking to one of Ziervogel et al.’s [44] three central aspects of transformational capacity, namely social cohesion. The second central aspect, one’s own agency, is also evident in Wurrumiyanga and many other rAcs [54]. The power of voice, individual knowledge and local knowledge shown by the IL and IR groups in step 1 of the frame analysis [29] showed that both agency and social cohesion are present and underpin and support the third aspect, reconnection of natural and man-made life support systems that support daily well-being [53].

Two of the feedback loops were not fully discussed. In common with the frame analysis [29], it was observed that some IL group members, although interested in the topic, did not want to spend a lot of time on their responses. Butler et al. [55] stated that maintaining participation is important to achieve the desired outcomes and impacts, however this depends on the mode of engagement. There is also the risk of consultation and participation fatigue if processes are not well developed, or if the participants feel that they are getting little reward or have no influence on the decision being made that will ultimately affect them [56]. Future research in rAcs must take this into account and be aware that providing more time to carry out the tasks will not necessarily ensure that they are completed.

The FGDs were difficult to initiate in Wurrumiyanga. The intention had been to run a full day workshop and multiple FGDs in parallel. However, it was challenging to organise and would have required participants to have given a whole day of their time. Eventually, it was decided to run separate FGDs, which were voluntarily divided by gender at the convenience of the participants and at a location where they felt comfortable over a 2-week period.

It is notable that as a result of this revised approach, the local participants felt at ease discussing difficult and complex issues. Before any component of the research had begun, the lead author had visited the community several times as a volunteer on veterinarian visits, followed by components of previous studies, and local community members were familiar with the individual. In addition, local community members expressed that they did not view the lead author as an “outsider”, but instead as someone who wished to help the community become healthier and who was trusted to the point that the author had transitioned from “coming amongst” to “coming alongside” the research subjects [54]. Martin’s [54] work on appropriate community engagement in rAcs, “Knocking Before You Enter”, is clearly equally important when working on companion animal management and in achieving meaningful data collection and collective action.

However, even with this approach, it was still impossible to set up an FGD with the fourth stakeholder group (NIL), because they were not available in the same place at the same time. This reflected similar challenges faced by Butler et al. [55] in Papua New Guinea while conducting multistakeholder livelihood planning: attendance by government and private sector stakeholders was poor, whilst participation by community members and local NGOs was enthusiastic. The omission of the NIL’s knowledge as a result is a limitation that needs to be considered.

Another factor determining the FGD methodology and participation was the fact that during the 2-week period when the FGDs were held, the lead researcher was introduced to groups that were already formed for other activities (e.g., men’s, women’s and elder’s groups), and therefore a choice was not always given as to who participated in any FGD. Future research initiatives should not assume that the same ILs will be available or willing to participate in successive components of research. This is also the case for NILs and AMs, particularly those working in remote communities, largely due to the high turnover of staff in public service positions. Animal management is not a primary portfolio for any level of government in the Northern Territory, and therefore it becomes overlooked and under-resourced. This is also a systemic cause of the dog overpopulation problem, as identified by the AM group during the frame analysis [29]. Similar reasons have been cited as the causes of under-representation of government stakeholders in multistakeholder adaptation planning [25].

## 5. Conclusions

When tackling complex problems involving multiple stakeholder groups, participatory methods such as CLA can enable all participants to contribute their knowledge, which is an important principle for addressing wicked problems. This study is interesting because despite their different frame analysis profiles, all Wurrumiyanga stakeholder groups involved in animal management agreed on the priority solution themes, namely “training” and “education”, and these were all transformational. Although the methodology was constrained by time and engagement limitations, which are typical of rAcs, overall the CLA process was accepted and undertaken with commitment by all involved. It was successful in so far as transformational actions were co-developed by all FGDs, which may have also built capacity and agency amongst the local community to implement them as a cohesive group. This laid the foundation for the following step in the Comm4Unity framework, designing stakeholder collaborations for impact. This methodology may not only assist in dog overpopulation, but may also guide further research in companion animal management, as well as other natural resource management studies across multiple disciplines and across multiple stakeholder groups. Its delivery within a remote Aboriginal community may also offer assistance in research in these areas.

## Figures and Tables

**Table 1:** Numbers, stakeholder groups and genders of participants involved in 10 focus group discussions (FGDs) considering the issue of “too many dogs” in the remote Aboriginal community of Wurrumiyanga, Tiwi Islands.

FGD	Stakholder Group	Participants	Male	Female	Number of Interviewees *
1	IL	4	50%	50%	1
2	IL	4	0%	100%	0
3	IL	5	0%	100%	0
4	IL	5	0%	100%	0
5	IL	4	0%	100%	0
6	IL	4	25%	75%	0
7	IL	26	100%	0%	0
8	IL	10	100%	0%	0
9	IR	3	100%	0%	2
10	AM	4	25%	75%	3

* Participants that had previously been interviewed in the frame analysis [31].

**Table 2:** Combined solutions identified by the 10 focus groups that participated in the causal loop analyses.

Rank (Stakeholder Group)	Solution	Stakeholders	Indicator of Success	Next Step
Training				
1 (IL)	More training for between vet visits and to help vet	TIRC	More people trained in animal management, parasite medicine delivery	Approach traditional owners to ask the TIRC to undertake more animal management training
1 (IL)	More training for young local people in animal management, which will improve animal health	Tiwi Islands Training and Employment Board (TITEB)	Improved dog health can be measured during routine census conducted	Attend skin group meeting and suggest that TITEB deliver animal management training
1 (IR)	Engage local people to contribute to animal management	TITEB	More people trained will result in healthier animals	TLC to ask TITEB for training
2 (IL)	Train local people how to keep dogs healthy	TIRC	Dogs become healthier and live longer	Send request to TIRC to ask vet to train people how to give medicine.
2 (AM)	Train local people in dog parasite control	Animal Management in Rural and Remote Indigenous Communities (AMRRIC), vet, researchers—TLC	More people trained in the delivery of parasite control	Identify who to train Secure storage, vehicles staff, supportive business structure, funding
Education				
1 (IL)	To introduce dog ownership and responsibility education into schools	TIRC	Dog ownership education classes begin at schools	Send request to TIRC to ask vet to help teach at school
1 (AM)	Deliver “responsible dog ownership” to empower local people	AMRRIC	Participant numbers, uptake of services, census	AMRRIC to consult with Tiwi schools and men and women groups to introduce responsible ownership education
2 (IR)	Teach owner responsibility for de-sexing to limit dog numbers	School via IRs	Dogs managed better, less dogs roaming, healthier dogs	Approach school (Principal) and ask if indigenous rangers can teach animal (dog/cat/pig) responsibilities at school
3 (IL)	Increase education about responsible dog ownership	School		Approach school (Principal) and ask if dog ownership responsibilities can be taught at school
Farming				
1 (IL)	We can create a local produce shop so we can buy food cheaper	Skin group meetings and traditional owners	A running shop would mean more money for locals	Approach traditional owners and ask them to take this to the skin group meeting
2 (IL)	Start farms on Tiwi (traditional and non-traditional) to provide local food and avoid expensive imported supermarket food	TITEB and/or school	Increase employment, healthier diets, cheaper food	Recommend to TITEB (for adults) and school (for children) to train or teach how to grow food
2 (IL)	Start farms to grow our own food	Elders	Producing food for eating and selling	Engage elders who remember and teach the children to grow gardens and plants traditionally eaten by Tiwi People
By-Laws				
1 (IL)	Introduce 2-dog by-law to reduce number of dogs per household	TIRC	Conduct census (UNE/AMRRIC) to count numbers, commence dog registrations	Chair of skin group meeting to follow up with the TIRC and mayor as this has already been started at skin group level.
1 (IL)	Create and/or enforce rules about not transporting dogs to Tiwi via plane and/or ferry	TIRC	Reduce the number of dogs brought to Tiwi	Attend skin group and suggest rules for dog transport and then take to TIRC
3 (IR)	New laws	TIRC and TLC	Less dogs	Discuss at skin group meeting then take to TIRC
Night Patrols/Neighbourhood Watch				
1 (IL)	Increase neighbourhood watch and night patrols	Police	Less reports of prowlers	Ask a traditional owner to ask the police for more neighbourhood watch
3 (IL)	More patrols at night time	Police and night patrol	Increase patrols = decease in prowlers	Attend skin group meeting and suggest going to police and asking for more night patrols
Other				
1 (IL)	Get dog pound up and running. Lock up roaming dogs—if not collected then remove	TIRC	Pound working and reduced stray dog numbers	Ask TIRC to finish the construction of the pound and to start utilising it
2 (IL)	Place cement under fences to stop dogs digging to escape and roam around town	Bathurst Island Housing Association	Conduct transect drives around the community to count the number of roaming dogs	Community elder to take idea to skin group meeting to move forward
3 (AM)	Consult experts during discussions e.g., when discussion by-laws, infrastructure (animal management plans)	TIRC—AMMRIC, vets, researchers	Number of incidents, development of agreements e.g., vet visit increase, funding, registration	Researchers to give advice to consult experts Experts should lobby/advertise that they are available

**Table 3:** A summary of the proposed solutions showing the frequency and priority (priority 1 = P1; priority 2 = P2; priority 3 or 4 = P3) for each stakeholder group. The totals for each theme across groups and their split between transformative and incremental solutions are also shown.

	IL			IR			AM			Total (Transformational/Incremental)
Theme/Priority	P1	P2	P3	P1	P2	P3	P1	P2	P3	
Training	2	1		1				1		5 (5/0)
Education	1		1		1		1			4 (4/0)
Farming	1	2								3 (3/0)
By-laws	2					1				3 (2/1)
Night patrol	1		1							2 (2/0)
Vet visits		1				1				2 (2/0)
Pound	1									1 (0/1)
Cement fences		1								1 (1/0)
Expert consults									1	1 (1/0)
